# Perturbations of mesenchymal stromal cells after allogeneic hematopoietic cell transplantation predispose for bone marrow graft-versus-host-disease

**DOI:** 10.3389/fimmu.2022.1005554

**Published:** 2022-10-12

**Authors:** Thomas Krüger, Rebekka Wehner, Maik Herbig, Martin Kräter, Michael Kramer, Jan Moritz Middeke, Friedrich Stölzel, Catrin List, Katharina Egger-Heidrich, Raphael Teipel, Uta Oelschlägel, Martin Wermke, Helena Jambor, Manja Wobus, Johannes Schetelig, Korinna Jöhrens, Torsten Tonn, Julien Subburayalu, Marc Schmitz, Martin Bornhauser, Malte von Bonin

**Affiliations:** ^1^ Department of Internal Medicine I, University Hospital Carl Gustav Carus, Dresden, Germany; ^2^ German Cancer Consortium (DKTK), Partner Site Dresden, and German Cancer Research Center (DKFZ), Heidelberg, Germany; ^3^ Institute of Immunology, Faculty of Medicine Carl Gustav Carus, Technische Universität Dresden, Dresden, Germany; ^4^ National Center for Tumor Diseases (NCT), Dresden, Germany; ^5^ Max Planck Institute for Science of Light and Max-Planck-Zentrum für Physik und Medizin, Erlangen, Germany; ^6^ Biotechnology Center, Center for Molecular and Cellular Bioengineering Technical University (TU) Dresden Tatzberg, Dresden, Germany; ^7^ Center for Regenerative Therapies (CRTD), Dresden, Germany; ^8^ University Cancer Centrum (UCC), Early Clinical Trial Unit (ECTU), University Hospital Carl Gustav Carus, Dresden, Germany; ^9^ Institute of Pathology, University Hospital Carl Gustav Carus, Dresden, Germany; ^10^ Institute of Transfusion Medicine, Faculty of Medicine Carl Gustav Carus, Technische Universität Dresden, Dresden, Germany; ^11^ German Red Cross Blood Donation Service North-East, Dresden, Germany; ^12^ Mildred Scheel Early Career Center, Medical Faculty, Technische Universität Dresden, Dresden, Germany

**Keywords:** mesenchymal stromal cells, allogeneic hematopoietic stem cell transplantation, graft-versus-host-disease, alloreactivity, bone marrow niche

## Abstract

Functional impairment of the bone marrow (BM) niche has been suggested as a major reason for prolonged cytopenia and secondary graft failure after allogeneic hematopoietic cell transplantation (alloHCT). Because mesenchymal stromal cells (MSCs) serve as multipotent progenitors for several niche components in the BM, they might play a key role in this process. We used collagenase digested trephine biopsies to directly quantify MSCs in 73 patients before (n = 18) and/or after alloHCT (n = 65). For the first time, we demonstrate that acute graft-versus-host disease (aGvHD, n = 39) is associated with a significant decrease in MSC numbers. MSC reduction can be observed even before the clinical onset of aGvHD (n = 10). Assessing MSCs instantly after biopsy collection revealed phenotypic and functional differences depending on the occurrence of aGvHD. These differences vanished during *ex vivo* expansion. The MSC endotypes observed revealed an enhanced population of donor-derived classical dendritic cells type 1 and alloreactive T cells as the causing agent for compartmental inflammation and MSC damage before clinical onset of aGvHD was ascertained. In conclusion, MSCs endotypes may constitute a predisposing conductor of alloreactivity after alloHCT preceding the clinical diagnosis of aGvHD.

## Introduction

Allogeneic hematopoietic cell transplantation (alloHCT) has emerged as a powerful treatment option for various hematological indications. Nevertheless, its success is hampered by a considerable risk for life threatening infections and an allogeneic destruction of non-malignant recipient tissues by donor immune cells, termed graft-versus-host disease (GvHD) (1). Large clinical studies have identified GvHD as an independent risk factor for a delayed lymphatic recovery and a secondary failure of platelet recovery (2, 3). Moreover, a low platelet count was recognized as an independent risk factor in patients with GvHD (4, 5). Because hematopoietic stem cells (HSC) and its progeny are donor-derived, ineffective hematopoiesis as consequence of a direct immunological attack by donor immune cells appears to be unlikely. In this respect, a low platelet count might represent a more fundamental deregulation of the bone marrow’s (BM) homeostasis (5). In contrast, BM stromal cells (BMSC) remain recipient-derived and therefore may constitute the fundamental basis for alloreactivity. BMSC entail a heterogeneous population including endothelial cells (ECs), osteoblasts, fat cells, CXCL12 abundant reticular cells, and mesenchymal (stem) stromal cells (MSCs), which have been described to contribute to the hematopoietic niche (HPN) (6).

When estimated in BM aspirates of GvHD patients, BMSC quantities were reported in relation to either total nucleated cells (TNC) or CD45^+^ cells (6, 7). These results are systematically biased owing to the absolute number of hematopoietic cells predominant within the BM which is supposed to be reduced in GvHD compared to non-GvHD patients (8, 9) Furthermore, BM aspirates bear the risk of admixture of peripheral blood and do not completely cover all types of BMSC in the original composition.

To date, only a few clinical studies addressed the question how and to what extent BMSC are affected by GvHD based on BM sections: Mensen et al. observed increased numbers of BM-infiltrating T cells and reduced numbers of osteoblasts in patients with delayed B cell lymphopoiesis and aGvHD (10). It needs to be noted that the patient stratification in this specific study had been done according to B cell numbers obtained around day 90 after alloHCT, whereas BM sections had been obtained already 3–4 weeks after alloHCT. Time point of GvHD diagnosis as well as treatment have not been reported. In contrast, Shono et al. only observed a reduction of osteoblasts and B cells in patients with extensive chronic GvHD but not in the early phase after alloHCT (11). Medinger et al. focused on nestin^+^ perivascular niches. They reported a diminution of nestin^+^ cells during aGvHD, which returned to baseline after aGvHD had resolved (12). Unexpectedly, also an increased microvessel density in patients with aGvHD was oberved in this study, which is contradictory to several other studies reporting an EC damage related to GvHD (7, 12, 13). In conclusion, there are still uncertainties according to BMSC quantities that might be partly explained by methodological issues, focus on different types of BMSC, patient stratification and especially time point of BM examination in relation to clinical onset of GvHD, which was not consistently reported.

Since MSCs give rise to several other non-hematopoietic cells they serve as key players for HPN functions. Herein, we have used our recently published method for the isolation of MSCs from trephine biopsies. In brief, biopsies were collagenase digested at 37°C for 90 min (14). Using this approach we determined MSC quantities for the first time independent of BM cellularity in patients before and after alloHCT, without GvHD or in relation to clinical diagnosis of aGvHD (according to Harris et al.) (15). In addition to functional characterization of *in vitro* expanded MSC, directly isolated MSCs were studied without prior *ex vivo* expansion *via* multicolor flow cytometry (MFC). Using MFC for immune cell phenotyping, analysis of BM-infiltrating immune cells was undertaken to foster our understanding of the MSC-immune cell landscape concerning GvHD.

## Results

### Mesenchymal stromal cell quantity and size of fibroblastic colony-forming units coincide with moderate to severe acute graft-versus-host-disease

**Figure 1:**
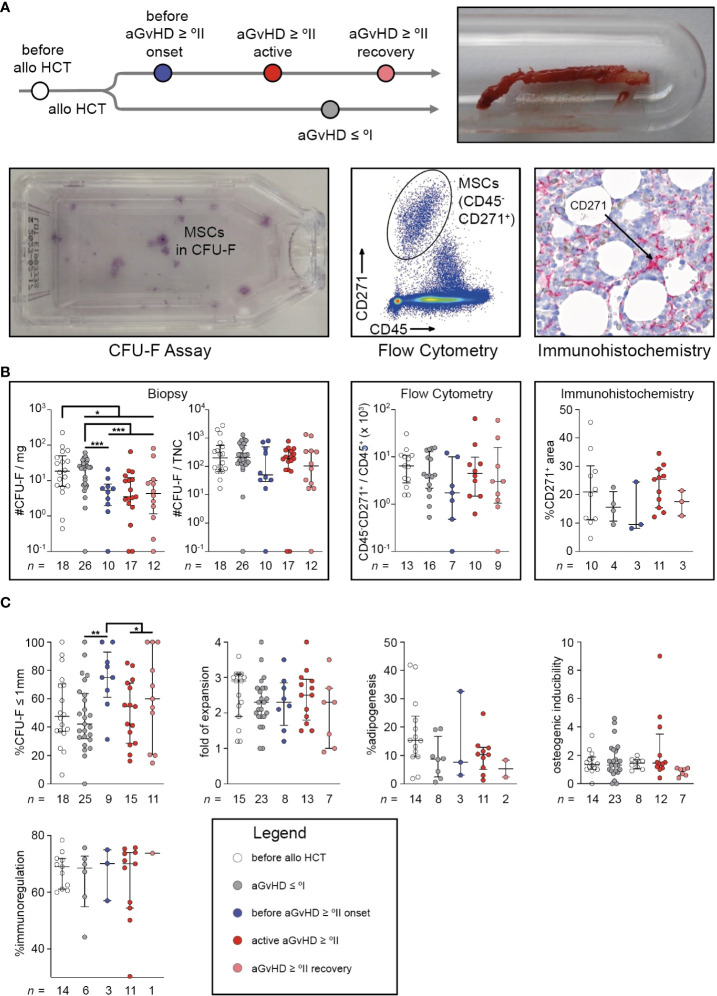
Quantity of mesenchymal stromal cells and their size of fibroblastic colony-forming units coincide with moderate to severe acute graft-versus-host-disease. **(A)** Study design: trephine biopsy specimens were obtained from routine diagnostics. After collagenase digestion, cells were seeded for CFU-F assay or stained to identify CD45^-^CD271^+^ cells using MFC, after gating on PI^−^CD235a^−^ single cells. Trephine biopsies from routine diagnostics were also used for immunohistological staining and calculation of CD271^+^ area (black arrows) related to total cellularized area. **(B)** CFU-F capacity related to the trephine biopsy’s weight and related to TNC, freshly isolated uncultured MSCs defined as PI^−^CD235a^−^CD45^−^CD271^+^ related to non-erythroid hematopoietic cells defined by PI^−^CD235a^−^CD45^+^ and quantification of MSCs using immunohistochemistry are shown. **(C)** The proportion of small colonies (≤1 mm) in CFU-F assays derived from MSC P0, population doubling time of MSC P1, the percentage of adipogenic different sites of MSC P2 layers, the osteogenic inducibility of MSC P2 layers (units of alkaline phosphatase of differentiated wells related to undifferentiated controls) and the immunomodulatory capacity of MSC P2 (T cell proliferation in cocultures with irradiated MSC P2 related to monocultures after anti-CD3/CD28 bead activation) are illustrated. The graphs show individual values with median and interquartile ranges. The Mann-Whitney U-tests were used for statistical analyses. n, number; *P < 0.05, **P < 0.01, ***P < 0.001.

Samples from patients before or after alloHCT were obtained. A detailed list of the patient’s characteristics is shown in **Table 1**. Patients after alloHCT were stratified according to the occurrence of aGvHD: 1) samples from patients who have *never* experienced aGvHD ≥ °II and 2) samples obtained *prior* to aGvHD ≥ °II onset, 3) *during* active phase of aGvHD ≥ °II, or 4) at least 30 days *after* last documentation of aGvHD ≥ °II (associated with a tapering of immunosuppressive therapy) (**Figure 1A**). Onset of aGvHD was defined as the day when symptoms of aGvHD ≥ °II were documented for the first time. GvHD severity was assessed and graded clinically according to Harris et al. (citation) (15).

**Table 1:** Patient characteristics.

All patients		n = 73
Age [y]	median (range)	58.9 (19.0 - 75.1)
Sex	female/male [n] (%)	22/51 (30.1/69.9)
Disease	AML [n] (%)	46 (63.0)
	MDS [n] (%)	15 (20.5)
	ALL [n] (%)	4 (5.5)
	MPN [n] (%)	4 (5.5)
	NHL [n] (%)	4 (5.5)
Patients with BM samples after alloHCT [n] (%)		60 (82.2)
Conditioning	RIC/MAC [n] (%)	42/18 (70.0/30.0)
	TBI ≥ 8 Gy [n] (%)	8 (13.3)
		
Graft	BM/PBSC [n] (%)	2/58 (3.3/96.7)
CD34^+^ [10^6^/kg]	median (range)	7.2 (1.7 - 16.5)
Donor	MSD [n] (%)	7 (11.7)
	MUD [n] (%)	40 (66.7)
	MMUD [n] (%)	9 (15.0)
	haplo [n] (%)	3 (5.0)
		
CMV (donor/recipient)	−/− [n] (%)	18 (30.0)
	−/+ [n] (%)	15 (25.0)
	+/+ [n] (%)	21 (35.0)
	+/− [n] (%)	6 (10.0)
		
GvHD prophylaxis	MTX + CSA/Tacro [n] (%)	49 (81.7)
	other [n] (%)	11 (18.3)
	+ATG [n] (%)	23 (38.3)
Patients with aGvHD [n] (% of patients after alloHCT)		46 (76.7)
aGvHD severity	°I [n] (%)	11 (23.9)
	°II [n] (%)	20 (43.5)
	°III [n] (%)	9 (19.6)
	°IV [n] (%)	6 (13.0)

aGvHD, acute graft-versus-host-disease; AL, acute leukemia; ALL, acute lymphatic leukemia; alloHCT, allogeneic hematopoietic stem cell transplantation; AML, acute myeloid leukemia; ATG, anti-thymocyte globulin; BME, bone marrow examination; CMV, cytomegalovirus; CSA, cyclosporine A; Gy, Gray; kg, kilogram; haplo, haploidentical; MAC, myeloablative conditioning; MDS; myelodysplastic syndrome; MMUD; mismatched unrelated donor; MPN, myeloproliferative neoplasm; MSD, matched sibling donor; MTX, methotrexate; NHL, non-Hodgkin lymphoma; PBSC, peripheral blood stem cells; RIC, reduced intensity conditioning; Tacro, tacrolimus; TBI, total body irradiation; y, year.

CFU-F capacity per milligram biopsy was significantly reduced after alloHCT (*P* < 0.05), especially in association with aGvHD (aGvHD ≥ °II versus aGvHD ≤ °I: *P* < 0.0001). Interestingly, this reduction had already occurred *before* aGvHD diagnosis (before aGvHD ≥ °II onset versus aGvHD ≤ °I: *P* < 0.001) (**Figure 1B**). In contrast, other typical MSC assessments in relation to cellularity (#CFU-F/TNC, CD45^-^CD271^+^/CD45^+^, and immunohistological quantification, i.e. %CD271^+^ of cellularized area) are systematically biased owing to the differing number of hematopoietic cells within the tissue samples and therefore incapable of reliably detecting changes of MSC quantities (**Figure 1B**). Only a slight and non-significant decrease in cellularity-dependent MSC numbers could be observed in samples taken *before* aGvHD which vanished during active aGvHD (**Figure 1B**).

As expected, the cohorts differed significantly in the time from alloHCT to BM sampling (median (min – max), aGvHD ≤ °I: 104 (20 – 800); before aGvHD ≥ °II onset: 71 (27 – 154); active aGvHD ≥ °II: 87 (29 – 230); aGvHD ≥ °II recovery: 334 (71 – 749)), which might have influenced the BM’s capacity for stromal regeneration. Furthermore, aGvHD-associated myelosuppression might have occurred without clinically evident aGvHD. However, in a multivariable analysis encompassing the variables time from alloHCT, platelet count, BM cellularity, and occurrence of aGvHD ≥ °II, only the appearance of aGvHD was significantly associated with reduction in MSC numbers (*P* < 0.001) (**Table 2**). When colonies of the CFU-F assay were categorized according to their size (≤ 1mm versus > 1mm), we observed a significantly higher proportion of small colonies before aGvHD onset compared with aGvHD ≤ °I (*P* < 0.01). This effect was attenuated *after* the onset of aGvHD (**Figure 1C**) and could also be seen in aspirates (**Figure S2**). However, MSCs in the first passage (P1) showed no differences in growth kinetics (**Figure 1C**). In passage 2 (P2), we observed a proclivity for a reduced adipogenic inducibility in patients after alloHCT (**Figure 1C**). There were neither differences in osteogenic inducibility nor immune-modulatory capacity in P2 (**Figure 1C**). We conclude that MSC numbers are significantly reduced in patients with aGvHD and that this decline is already detectable before the clinical appearance of aGvHD symptoms.

**Table 2:** Multiple linear regression analysis of CFU-F/mg.

	Estimate	(95% CI)	p-value
(Intercept)	3.982	(2.322 - 6.472)	0.000
Time from alloHCT [d]	-0.001	(-0.002 - 0.001)	0.342
Platelet count [Gpt/L]	0.001	(-0.003 - 0.004)	0.743
Cellularity (category: not reduced)	0.406	(-0.188 - 1.434)	0.219
aGvHD (category: aGvHD ≤ °I)	1.945	(0.707 - 4.082)	0.0002

### Phenotypic characterization of uncultured mesenchymal stromal cells

**Figure 2:**
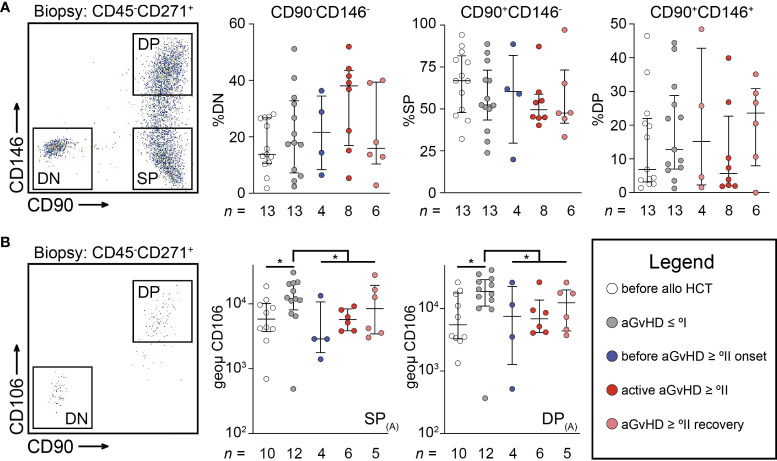
Uncultured mesenchymal stromal cells reveal dynamic endotypes after allogeneic stem cell transplantation. **(A)** Representative scatterplot showing three distinct endotypes of PI^−^CD235a^−^CD45^−^CD271^+^ cells (MSC) isolated from a trephine biopsy according to CD90 and CD146 expression. Here, frequency of MSC endotypes according to CD90 and CD146 expression are shown for double negative (DN: CD90^-^CD146^-^), single positive (SP: CD90^+^CD146^-^), and double positive (DP: CD90^+^CD146^+^), respectively. **(B)** Representative scatterplot showing the exclusive expression of CD106 within CD90^+^ MSCs from BM aspirates. The intensity of CD106 expression was separately analyzed on SP or DP endotypes as defined in **(A)**. Graphs show individual values with median and interquartile ranges. The Mann-Whitney U-tests were used for statistical analyses. n, number; *P < 0.05.

The changes pertaining to MSCs following alloHCT led us to investigate if MSCs can acquire different endotypes within the HPN that accrue to and orchestrate such changes culminating in aGvHD. Using MFC, several endotypes of uncultured MSCs (PI^−^CD235a^−^CD45^−^CD271^+^) could be identified within a CD90^+^ and a CD90^−^ fraction. CD271 is considered to be a pan-MSC marker (15, 16), whereas CD146 and CD90 only capture a fraction of the pan-MSC population. Consequently, isolated MSCs could be divided into three endotypes (**Figure 2A**): a double negative (DN: CD90^-^CD146^-^), a single positive (SP: CD90^+^CD146^−^), and a double positive endotype (DP: CD90^+^CD146^+^). We observed a trend toward less frequent expression of CD90 and CD146 in patients with *active* aGvHD ≥ °II (**Figure 2A**). We could not observe CD106 expression on biopsy-derived MSCs, an observation we attribute to the process of collagenase digestion (17, 18). Therefore, analysis of CD106 expression was restricted to MSC derived from BM aspirates. CD106 was found to be exclusively expressed within the CD90^+^ MSC endotype (**Figure 2B**). CD106 expression was significantly elevated in patients after alloHCT with aGvHD ≤ °I in both SP MSCs (CD90^+^CD146^-^) and DP MSCs (CD90^+^CD146^+^) endotypes alike (*P* < 0.05 for each comparison). Strikingly, CD106 expression was significantly downregulated on SP and DP MSC endotypes in patients with aGvHD ≥ °II (**Figure 2B**). Therefore, we suggest that differently expressed VCAM1 on MSC endotypes may propagate compartmental inflammation. We suggest that VCAM1 may act as an immunological activation signal.

### Functional *in vitro* characterization of mesenchymal stromal cells relating to homeostatic functions within the hematopoietic niche

**Figure 3:**
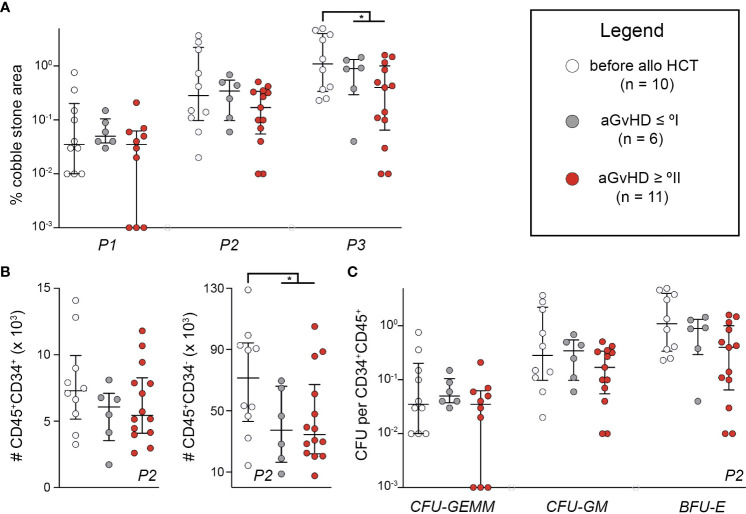
Endotypes of mesenchymal stromal cells relating to hematopoietic niche functions are lost during ex vivo expansion. **(A)** In CAFC test, cell layers were photographed using a Celigo S and scored for CAFC after 1, 2, and 3 weeks. Total area of CAFC of each coculture was calculated using ImageJ and compared with the total MSC layer area. **(B)** Additional cocultures of patient-derived MSCs P2 and healthy donor HSCs were detached after 2 weeks and quantified with a MACSQuant™ after staining for CD34 and CD45. **(C)** Detached cells from coculture were also used for CFC assay. CFUs were analyzed with STEMvision™. The number of CFUs in proportion to seeded CD34^+^CD45^+^ cells is shown. Graphs show individual values with median and interquartile ranges. The Mann-Whitney U-tests were used for statistical analyses. n, number; *P < 0.05.

To assess the impact of recipient MSC endotypes following alloHCT and in relation to aGvHD, abilities to support HSC function *in vitro* were tested by cobblestone-area forming cell (CAFC) test and subsequent colony-forming cell (CFC) assays. We compared MSCs from samples (i) *before* alloHCT, (ii) *after* alloHCT without aGvHD ≥ °II, and (iii) *after* alloHCT with aGvHD ≥ °II. Total area of cobblestones was determined after 1, 2, and 3 weeks. At 3 weeks, there was a significantly higher activity of cobblestone formation using MSCs from *before* alloHCT versus *after* alloHCT (*P* < 0.05) (**Figure 3A**). At 2 weeks, CD45^+^CD34^−^ and CD45^+^CD34^+^ were quantified using MFC. There were significantly more mature, i.e., CD34^−^CD45^+^ cells in samples based on MSCs collected *before* alloHCT versus *after* alloHCT (*P* < 0.05), although this observation could not be ascertained for immature, i.e., CD34^+^CD45^+^ cells (*P* > 0.05) (**Figure 3B**). There were no significant differences between aGvHD ≥ °II and aGvHD ≤ °I. Within CD34^+^CD45^+^ cells myeloid cell differentiation capacity was determined by the CFU assay (CFU-granulocyte, erythrocyte, monocyte, megakaryocyte [GEMM], CFU-granulocyte, monocyte [GM], and burst-forming unit-erythrocyte [BFU-E]), which did not differ significantly between any of the groups (**Figure 3C**). There was no correlation between the results obtained from CAFC or CFC data and the patient’s blood count or BM cellularity (data not shown). We conclude that alloHCT and the occurrence of aGvHD sparsely influence the functionality of *ex vivo* propagated MSCs as a consequence of plasticity to standardized microenvironmental conditions (19). Thus, we suggest that the *ex vivo* expansion of MSCs has mitigated any pre-existing differences on MSC endotypes and more tailored *in vitro* cultivation conditions need to be devised to maintain MSC endotype properties *ex vivo*.

### Bone marrow graft-versus-host-disease is linked to compartmental inflammation and priming of alloreactive T cells

**Figure 4:**
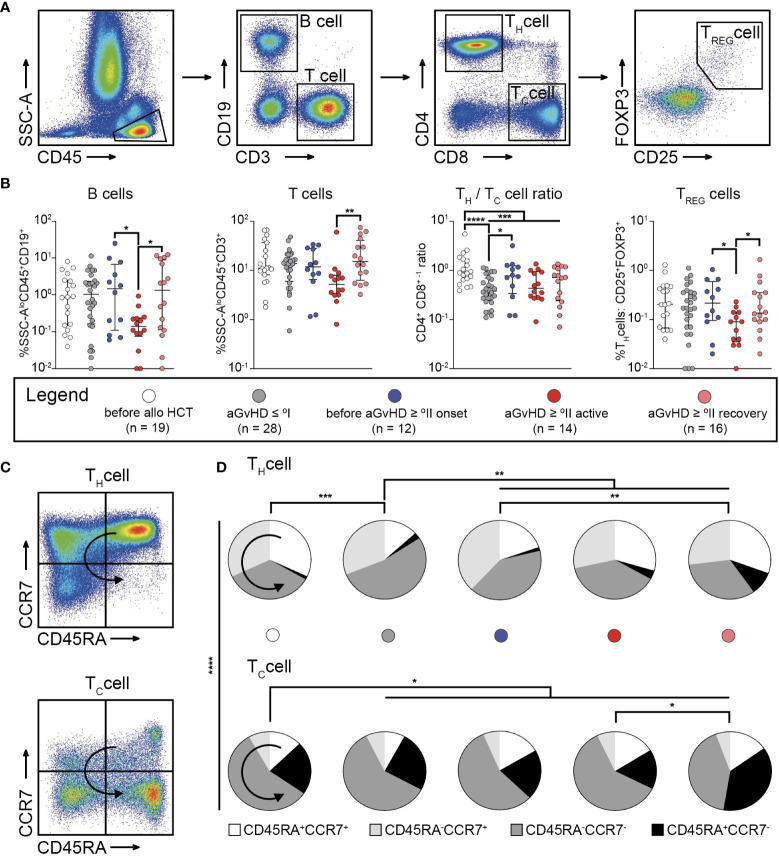
Bone marrow graft-versus-host-disease is linked to compartmental inflammation and priming of alloreactive T cells. **(A)** Gating strategy to identify B cells, and T cell subsets within SSC-A low (SSC-A^lo^)CD45^+^ cells in bone marrow aspirates. B cells were identified as CD3^-^CD19^+^ cells, T cells as CD3^+^CD19^-^ cells. T cells could be further subdivided into T helper (T_H_) cells (CD4^+^CD8^-^), cytotoxic T (T_C_) cells (CD4^-^CD8^+^), or regulatory T_H_ (T_REG_) cells (CD4^+^CD25^+^FOXP3^+^). **(B)** Results for the frequency of B cells, T cells, the CD4^+^ CD8^+ -1^ ratio, and frequency of T_REG_ cells identified within bone marrow aspirates from patients before allogeneic stem cell transplantation (alloHCT) (n = 19), and such following alloHCT either with aGvHD ≤ °I (n = 28), before onset of aGvHD ≥ °II (n = 12), with active aGvHD ≥ °II (n = 14), or having recovered from aGvHD ≥ °II (n = 16) are shown. **(C)** The gating strategy to identify memory or alloreactive subsets within CD4^+^ and CD8^+^ T cells according to the expression pattern of CD45RA and CCR7 are depicted. **(D)** Fractions of memory subsets within CD4^+^ and CD8^+^ T cells according to expression of CD45RA and CCR7 in BM aspirates from patients before and after alloHCT. Patients after alloHCT were further stratified according to the occurrence of aGvHD. Graphs show individual values with median and interquartile ranges or pie charts **(D)**, respectively. The Mann-Whitney test was used for statistical analysis; *P < 0.05, **P < 0.01, ***P < 0.001, ****P < 0.0001; ↪ direction of differentiation.

MFC data of BM aspirates concerning lymphoid cells with a focus on T cell subpopulations (**Figure 4A**) revealed a significant reduction of B cells and regulatory T helper (T_REG_) cells in patients with *active* aGvHD ≥ °II compared with those in patients *before* aGvHD ≥ °II onset and *after* aGvHD (*P* < 0.05) (**Figure 4B**). There were no significant differences in the proportion of T_REG_ cells and B cells between patients before alloHCT, after alloHCT with aGvHD ≤ °I, *before* occurrence of aGvHD ≥ °II, and *after* aGvHD ≥ °II, respectively. Frequency of T cells was higher in patients *after* aGvHD ≥°II (*P* < 0.01) (**Figure 4B**). The CD4^+^/CD8^+^ ratio was generally reduced after alloHCT (*P* < 0.001). Patients with aGvHD ≤ °I were characterized by the lowest CD4^+^/CD8^+^ ratio following alloHCT (*P* < 0.0001). Compared to patients with aGvHD ≤ °I, patients *before* onset of aGvHD ≥ °II displayed an elevated CD4^+^/CD8^+^ ratio (*P* < 0.05) (**Figure 4B**). Since T_H_1 cells are potent sources of IFN-γ and TNF-α, we regarded T_H_1 cell frequencies as a proxy for these cytokines within the bone marrow. Hence T_H_1 cell frequencies were used to indicate compartmental inflammation and potentially alloreactivity directed against recipient-derived MSCs (19, 20). Hence, we sought to analyze CD4^+^ and CD8^+^ T cells (T helper (T_H_) or cytotoxic T (T_C_) cells, respectively) regarding their memory and activation phenotype by staining for CD45RA and CCR7 (**Figure 4C**). After alloHCT, we observed a significant shift from quiescent to more activated T cell subsets both in the CD4^+^ and the CD8^+^ fraction (**Figure 4D**). Within CD4^+^ T cells, this effect was most pronounced in patients devoid of aGvHD, evidenced by a significant decrease of CD45RA^+^CCR7^+^ naïve (*P* < 0.001) and an increase of CD45RA^−^CCR7^−^ T effector memory (T_EM_) cells (*P* < 0.01). Compared with patients devoid of aGvHD, patients with aGvHD ≥ °II displayed an elevated frequency of CD45RA^+^CCR7^+^ T cells (*P* < 0.01), which harbour a subset that differentiate directly from naive T cells upon TCR engagement and retain the capacity of self-renewal and to hierarchically differentiate into all other memory T-cell subsets, called TSCM (21). CD45RA^−^CCR7^−^ T_EM_ cells were significantly decreased in this cohort (*P* < 0.01). Additionally, we observed an increase of terminally differentiated CD4^+^ T helper (T_H_) cells in the course of aGvHD (*before* aGvHD ≥ °II onset versus *after* aGvHD ≥ °II: *P* < 0.01). CD8^+^ T cells generally displayed a more differentiated/activated phenotype. There was a slight but significant decrease of CD45RA^−^CCR7^+^ after alloHCT independent of the occurrence of aGvHD occurrence (*P* < 0.05) and an increase of terminal effector cells *after* aGvHD (*P* < 0.05) (**Figure 4D**).

**Figure 5:**
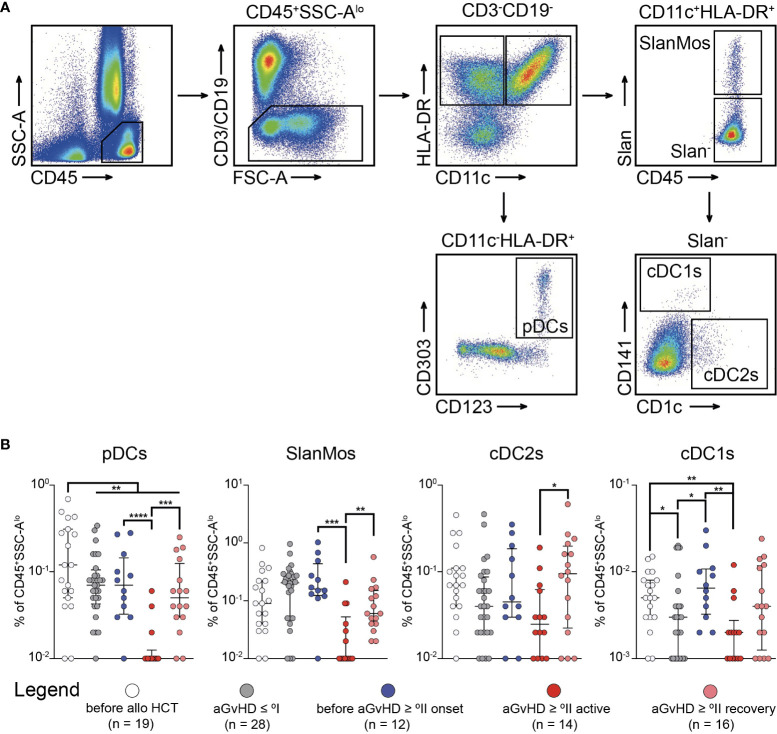
Infiltration of CD141^+^ dendritic cells occurs prior to active acute graft-versus-host-disease ≥ °II and is depleted with immunosuppressive treatment. **(A)** The gating strategy to identify subpopulations of dendritic cells (DCs) in bone marrow (BM) aspirates is shown. CD45+SSCloCD3−CD19−HLA-DR+ cells were further characterized as plasmacytoid DCs (CD11c−CD123+CD303+) (pDCs), Slan-positive monocytes (CD11c+Slan+) (SlanMos), type 1 classical DCs (CD11c+Slan−CD141+CD1c−) (cDC1s), and type 2 classical DCs (CD11c+Slan−CD141−CD1c+) (cDC2s). **(B)** Frequency of DC subpopulations in percentage of CD45+ cells in BM aspirates. Graphs show individual values with median and interquartile ranges. The Mann-Whitney test was used for statistical analysis; *P < 0.05, **P < 0.01, ***P < 0.001, ****P < 0.0001.

Having observed the enriched population of activated T_H_ cells (CD45RA^-^CCR7^+^) preceding the clinical diagnosis of moderate to severe BM-GvHD, we sought to investigate whether this patient cohort could be characterized any predisposing factor driving alloreactive T cell priming. Previous research has shown that compartmental inflammation can cause functional impairment and lack of longevity in MSCs (20–22). Consequently, necrotic MSCs might be sensed by classical DCs, which are capable to cross present MSC-derived antigens to T cells. Ultimately, T cell priming occurs (23, 24), which increases the likelihood for BM-GvHD. Thus, we sought to characterize the myeloid compartment within the BM with a particular focus on DCs (**Figure 5A**). Regarding the frequency of DCs, we observed a clear reduction of plasmacytoid DCs (pDCs) after alloHCT independent of the occurrence of aGvHD (*P* < 0.01) with an additional diminution during *active* aGvHD ≥ °II (versus *before* aGvHD ≥ °II: *P* < 0.0001 and versus *after* aGvHD ≥ °II: *P* < 0.001) (**Figure 5B**). Instead, 6-sulfo LacNAc-positive Monocytes (SlanMos) showed an increase after alloHCT possibly reflecting an inflammatory state but were also markedly diminished during *active* aGvHD ≥ °II (versus *before* aGvHD ≥ °II: *P* < 0.001 and versus *after* aGvHD ≥ °II: *P* < 0.01) (**Figure 5B**). For CD141^-^CD1c^+^DCs (cDC2s), we made similar observations as for pDCs. cDC1s exhibited the lowest frequencies among all DC subpopulations. The background of alloHCT led to a significant reduction in cDC1s (before alloHCT vs. aGvHD ≤ °I: *P* < 0.05). However, patients *before* aGvHD ≥ °II were characterized by an enhanced frequency of cDC1s compared with that of patients with aGvHD ≤ °I (*P* < 0.05) similar to the level observed before alloHCT (*P* > 0.05). Patients with *active* aGvHD ≥ °II showed the lowest CD141^+^DC frequency of all cohorts (*P* < 0.01 compared with patients *before* aGvHD ≥ °II). The frequency gradually increased again in patients having recovered from active aGvHD, which we attribute to a tapering of the immunosuppressive medication.

## Discussion

Destruction of the BM stroma is conceived to be accountable for aGvHD-associated myelosuppression (6, 7, 10, 11, 13, 20–22, 25). Previous clinical studies uniformly reported a disproportionate reduction of stromal cells compared with hematopoietic cells in BM aspirates (6, 7, 20). Our data suggest that this reduction in recipient derived MSCs occurs even prior to clinical onset of aGvHD > II°. We also observed that this compartmental inflammation coincided with an enriched fraction of cDC1s and alloreactive T_H_ cells as the potential source for MSC-directed destruction of the HPN.

Processing BM biopsies by collagenase digest revealed a reduction in recipient derived MSCs even prior to the onset of clinical aGvHD. Additionally, this reduction was associated with smaller MSC colonies using the CFU-F assay. Using BM aspirates we could not validate this finding. This is because calculating MSC numbers in relation to the specimen’s weight is less prone to systematic bias linked to differing total BM cellularity between patient cohorts and random bias due to blood admixture as in BM aspirates employed routinely. Instead, the use of collagenase-digested trephine biopsies enables MSC quantification independent of BM cellularity and peripheral blood admixture. However, steroid treatment of patients with ongoing aGvHD might have influenced MSC quantitites. Conversely to previous studies focusing on osteoblasts and nestin^+^ perivascular cells, our study revealed no recovery of the HPN after aGvHD had resolved (10, 11, 25). In accordance with a recent animal study that reported no recovery of CFU-F activity after injury to the HPN had occurred (26), multivariable analysis of our data revealed that time had no significant association with recovery of CFU-F capacity.

However, it should be noted that all results in relation to BM cellularity revealed a trend to reduced MSC frequencies only in the cohort *before* clinical onset of aGvHD. The lack of a significant reduction in relative MSC numbers during *active* aGvHD ≥ °II could be explained by a delayed decrease in BM cellularity. Together, this reduction in MSC numbers suggested subclinical aGvHD (“niche GvHD”) with a subsequent loss in immunoregulation *via* MSCs further promoting the onset of active aGvHD (“GvHD fuel”). The existing data on functional characterization of *ex vivo* expanded MSCs in the context of alloHCT are contradictory (20, 26, 27). In our study, we still observed an attenuated growth behavior of MSCs in P0 from patients *before* aGvHD ≥ °II. However, at later passage numbers, the functionality of *ex vivo* propagated MSCs was minimally influenced by alloHCT and aGvHD contradicting to previous reports. Nevertheless, pre-existing functional differences that still existed *in vivo* might be mitigated by the cultivation process *in vitro* (19).

To avoid cultivation-associated bias, we characterized uncultured MSCs regarding their expression of CD90, CD106, and CD146 using MFC. CD90 expression on MSCs was suggested to define stromal cells with high CFU-F capacity, greater differentiation and immunomodulatory potential (16, 28). Our data suggest that CD90^+^ MSCs behave differently with regard to CD106 (VCAM-1) and CD146 expression. *VCAM-1*, despite being a direct target gene of the nuclear factor of kappa B (NF-κB) and resultingly involved in mediating inflammatory processes (29, 30), was also reported to enhance engraftment of HSCs and their progeny (31–33). Indeed, VCAM1’s biological function may depend on whether it occupies its membrane-bounded or shedded form (33–35). As such, VCAM1^+^ MSCs can potently suppress activated T cell responses (34, 36–38), whereas VCAM1’s shedded form is linked to compartmental inflammation *via* TNF-α and IFN-γ. We have found a higher expression of VCAM1 on MSCs following alloHCT. Intriguingly, MSCs from patients prior to the onset of clinical aGvHD ≥ °II were characterized by reduced VCAM1^+^ MSCs, irrespective of co-expression of CD146 suggestive of nhanced shedding of VCAM1 and/or already ongoing destruction of VCAM1 expressing MSC. As such, a decrease in immunomodulatory capacity of MSCs can be surmised. However, further studies are required to assess the differential expression kinetics of VCAM1+ MSCs in the context of soluble VCAM1 within the BM. Moreover, T cell functionality assessments (TNF-α and IFN-γ production) of isolated T cell from BM aspirates of our patient cohorts should be performed upon recombinant human VCAM1 treatment (soluble effect) as opposed to isolated irradiated VCAM1^+^ MSCs in ex vivo tests.

We also investigated changes to the immune cell-MSC interface in relation to the observed changes in MSC endotypes. Here, we observed a strong reduction of immune cells including B cell, T_REG_ cells, and distinct DC subsets in BM aspirates of patients with aGvHD, as described earlier for peripheral blood or tissue sections (10, 11, 39, 40). Compared to before alloHCT, the lower T_H_/T_C_ cell ratio in all groups of patients after alloHCT is a well-known phenomenon and can be explained by a faster homeostatic peripheral expansion of T_C_ cells compared to that of T_H_ cells (10, 40, 41). Importantly, before clinical onset aGvHD ≥ °II, an enhanced ratio of T_H_/T_C_ cells was observed implying enhanced levels of the canonical TH cell cytokines TNF-α and IFN-γ within the BM. Jointly with a reduced immunomodulatory function of VCAM1^+^ MSCs, this led us to believe these T_H_ cells to be target of alloreactive priming from a naïve precursor with MSC-directed origin. Indeed, infiltration of target organs by alloreactive T cells is a key observation in aGvHD (1). GvHD patients are characterized by an enhanced fraction of fit CCR7^+^ T_H_ cells, which may predispose as the inducing agent for MSC-directed damage of the HPN. CCR7 endows T cells with a unique capability to home to lymph nodes *via* CCL19 and CCL21 (42). Alternatively, an enriched fraction of CCR7 could be found resulting from the destruction of the lymphatic system from the preconditioning therapy. Hence, CCR7^+^ naïve T cells preferentially enrich within the BM, which is rich in CCL19 (43). This is the place where their priming towards minor antigens and recipient-derived peptides from recipient-derived MSCs may be acquired. Interestingly, the general reduction in immune cells occurred almost exclusively during *active*/*ongoing* aGvHD but did not precede the clinical appearance of aGvHD ≥ °II. Several factors may contribute to these findings. The reduced amount of B cells during active aGvHD might represent a delayed consequence of the disturbance of the HPN. Further studies are needed to validate these observations. For example, the kinetics of CCL19/CCL21 expression should be set in relation to CCR7^+^ T cell homing to the BM. Efforts should be made to better firm up the interaction between CCR7^+^ naïve T cells and their ability to be primed with MSC antigens by CD11c^+^Slan^−^CD141^+^CD1c^−^ cDC1 cells in ex vivo assays.

Concerning DCs, their fraction in BM aspirates might have been additionally altered by migration of these cells to aGvHD target organs. This has been demonstrated for SlanMo which are supposed to exhibit a predominantly proinflammatory phenotype (44). Regarding pDCs, there is little evidence to support this hypothesis. Although identified as a main source of type I interferons and implicated in the development of many inflammatory diseases including aGvHD (45, 46), they have also been described as inducers of peripheral tolerance (39, 47, 48). Importantly, side effects of steroid treatment should be considered to contribute to alterations of immune cells during aGvHD: i) direct toxicity on the analyzed immune cells (49, 50), and ii) indirect toxicity mediated by pertubations of MSCs and their progeny (50, 51). We could observe that patients prior to the onset of aGvHD ≥ °II harboured an enhanced fraction of cDC1s. cDC1s are known to be capable of cross-presenting necrotic antigens or such derived from exosome-shedding to alloreactive T cells (27, 52, 53). Strikingly, these were also depleted with treatment during active aGvHD ≥ °II. We hypothesize these cDC1s as the inducing agent of alloreactive, MSC-directed T_H_ from naïve T_H_ cells by which the destruction of the HPN prior to clinical/active aGvHD ≥ °II occurs, and by which in the following peripheral cytopenia is deteriorated.

In conclusion, quantification of MSCs independent of BM cellularity from collagenase-digested trephine biopsies revealed that MSCs are diminished in association with aGvHD. This damage occurs prior to the clinical onset of aGvHD and is not reversed after recovery from aGvHD. It is noteworthy that the clinically observed myelosuppression appears in direct temporal association with aGvHD diagnosis in most cases. But even the lack of association between collected HPN parameters (MSC numbers, phenotype, and function) and peripheral blood counts in our cohort does not exclude a relevant contribution of dysfunctionality within the HPN to aGvHD-associated myelosuppression. We assume that a reduction in MSCs (or a derailed composition of MSC subtypes) might serve as a surrogate for imminent aGvHD. However, we acknowledge the inherent limitation of *in vivo* human studies. Especially, further studies are needed to address compartmental inflammation based on kinetic cytokine profiles of TNF-α, IFN-γ, IL-6 and TGF-β following alloHCT and in relation to aGvHD status. Arguably, contribution of T cell activation to alloreactivity needs to be further validated in ex vivo assays using MSC-conditioned DC cells.

## Materials and methods

### Patient and sample characteristics

BM aspirates and/or BM biopsy specimens were collected from routine diagnostic procedures from 73 hematologic patients undergoing alloHCT (**Table 1**). Samples with evidence of medullary relapse defined by the presence of ≥ 10% blasts in the case of acute leukemia/myelodysplastic syndrome/myeloproliferative neoplasm or any infiltration of non-Hodgkin lymphoma were excluded. Aspirates were available from 12 patients before, 46 patients after and 7 patients before and after alloHCT. Trephines were available in 13 patients before, 47 patients after and 7 patients before and after alloHCT. The study (NCT02829216) has been approved by the Institutional Review Board (EK 108032016).

### Isolation and quantification of mesenchymal stromal cells

BM samples were prepared as previously described (14). Collagenase-digested trephine biopsies were used for functional quantification of MSCs by the colony-forming unit fibroblast (CFU-F) assay and to detect uncultured MSC by MFC. Numbers of CFU-Fs divided by TNC resulted in relative MSC frequencies. CFU-F capacity per milligram biopsy provides results independent from BM cellularity. MSCs were defined by MFC as PI^−^CD235a^−^CD45^–^CD271^+^ and were normalized to the total leucocyte count (PI^−^CD235a^−^CD45^+^). In BM sections, MSC were quantified as CD271^+^ visual areas in relation to the total cellularized area. Detailed descriptions of the methodologies, equipment, reagents, and an example of the MFC gating strategy are provided in the **Supplemental Data**.

### Characterization of mesenchymal stromal cells

*In vitro* cultivation and characterization of MSC was performed as described previously (14). The phenotype of freshly isolated MSC without prior *in vitro* expansion was analyzed by MFC. For details see **Supplemental Data**.

### Characterization of immune cells residing within the bone marrow

Immune cells were analyzed using MFC in BM aspirates. Reagents are provided in the **Supplemental Data**. Examples of the gating strategies are shown in the results section.

### Statistical analyses

Statistical methods are provided in the **Supplemental Data**. All values are presented as median ± interquartile range. The Mann-Whitney *U* test was used to detect statistically significant differences between two or more unpaired groups. A *P* < 0.05 was considered statistically significant. GraphPad Prism 9.0 (GraphPad Software, Inc., California) was used for statistical analyses.

## Data availability statement

The original contributions presented in the study are included in the article/**Supplementary Material**. Further inquiries can be directed to the corresponding author.

## Ethics statement

This study was reviewed and approved by the local ethics committee of the University Hospital Carl Gustav Carus Dresden, Germany (EK 108032016). All subjects gave written informed consent in accordance with the Declaration of Helsinki. The patients/participants provided their written informed consent to participate in this study.

## Author contributions

TK designed research, performed research, collected data, analyzed, and interpreted data, performed statistical analyses, wrote the manuscript. RW performed research, collected data, analyzed, and interpreted data. MH, MaK, and MiK contributed vital new reagents or analytical tools. MiK performed statistical analyses. JM, FS, CL, KE-H, and RT performed research and collected data. UO performed research, collected data, and contributed vital new reagents or analytical tools. MWe performed research. HJ analyzed and interpreted data. MWo contributed vital new reagents or analytical tools. JoS and KJ performed research and collected data. TT contributed vital new reagents or analytical tools. JuS analyzed and interpreted data, performed statistical analyses, and wrote the manuscript. MS and MB designed research and wrote the manuscript. MvB designed research, performed research, analyzed, and interpreted data, and wrote the manuscript. All authors contributed to the article and approved the submitted version.

## Funding

The project was funded by the Deutsche Forschungsgemeinschaft (Clinician Scientist position to TK; a Seed grant within the Centre for Regenerative Therapies Dresden, www.crt-dresden.de; DFG Project number 399422891 to MH). JuS was funded by the Deutsche Forschungsgemeinschaft (Clinician Scientist position, SU 1360/1-1).

## Acknowledgments

We thank Katrin Müller, Claudia Richter, and Robert Kuhnert for performing MSC isolation and functional analyses assays of cultured MSCs. The authors would like to thank Enago (www.enago.com) for the English language review.

## Conflict of interest

The authors declare that the research was conducted in the absence of any commercial or financial relationships that could be construed as a potential conflict of interest.

## Publisher’s note

All claims expressed in this article are solely those of the authors and do not necessarily represent those of their affiliated organizations, or those of the publisher, the editors and the reviewers. Any product that may be evaluated in this article, or claim that may be made by its manufacturer, is not guaranteed or endorsed by the publisher.
