# Supplementary material for: Perturbations of mesenchymal stromal cells after allogeneic hematopoietic cell transplantation predispose for bone marrow graft-versus-host-disease
Source: Front Immunol. 2022 Oct 12;13:1005554. doi: 10.3389/fimmu.2022.1005554 (PMC9599394; doi:10.3389/fimmu.2022.1005554)
Supplement: Supplementary file 1 [file DataSheet_1.docx]

Supplementary Material to Perturbations of mesenchymal stromal cells after allogeneic hematopoietic cell transplantation predispose for bone marrow graft-versus-host-disease

**Thomas Krüger^1,2^, Rebekka Wehner^2,3,4^, Maik Herbig^5,6,7^, Martin Kräter^5,6^, Michael Kramer^1^, Jan Moritz Middeke^1^, Friedrich Stölzel^1^, Catrin List^1^, Katharina Egger-Heidrich^1^, Raphael Teipel^1^, Uta Oelschlägel^1^, Martin Wermke^1,8^, Helena Jambor^1^, Manja Wobus^1^, Johannes Schetelig^1^, Korinna Jöhrens^9^, Torsten Tonn^10,11^, Julien Subburayalu^1,2,7,12^, Marc Schmitz^2,3,4,7^, Martin Bornhauser^1,2,4,7^, Malte von Bonin^1,2^**

^1^Department of Internal Medicine I, University Hospital Carl Gustav Carus, Fetscherstr. 74, 01307 Dresden, Germany

^2^German Cancer Consortium (DKTK), Partner Site Dresden, and German Cancer Research Center (DKFZ), Heidelberg, Germany

^3^Institute of Immunology, Faculty of Medicine Carl Gustav Carus, Technische Universität Dresden, Dresden, Germany

^4^National Center for Tumor Diseases (NCT), Dresden, Germany

^5^Max Planck Institute for Science of Light & Max-Planck-Zentrum für Physik und Medizin, Erlangen, Germany

^6^Biotechnology Center, Center for Molecular and Cellular Bioengineering TU Dresden Tatzberg 47-49, Dresden, Germany

^7^Center for Regenerative Therapies (CRTD), Fetscherstr. 105, 01307 Dresden, Germany

^8^University Cancer Centrum (UCC), Early Clinical Trial Unit (ECTU), University Hospital Carl Gustav Carus, Fetscherstr. 74, 01307 Dresden, Germany

^9^Institute of Pathology, University Hospital Carl Gustav Carus, Fetscherstr. 74, 01307 Dresden, Germany

^10^Institute of Transfusion Medicine, Faculty of Medicine Carl Gustav Carus, Technische Universität Dresden, Dresden, Germany

^11^German Red Cross Blood Donation Service North-East, Dresden, Germany

^12^Mildred Scheel Early Career Center, Medical Faculty, Technische Universität Dresden, Dresden, Germany

*** Correspondence:**Thomas Krüger, M.D.
[thomas.krueger@uniklinikum-dresden.de](mailto:thomas.krueger@uniklinikum-dresden.de)

# Supplementary Methods

**Cell isolation from bone marrow aspirates**

Bone marrow aspirates were anticoagulated with EDTA and subjected to red blood cell lysis with ACK-Lysing Buffer (Gibco, Grand Island, New York, #A10492-01) according to the manufacturer’s instructions. The cell pellet was resuspended in phosphate-buffered saline (PBS) plus 5% fetal calf serum (FCS).

**MSC isolation from trephine biopsies**

Trephine biopsies were initially put in sterile 0.9% NaCl. Weighing was done in 1,5ml tubes (Sarstedt AG & Co. KG, Nümbrecht, Germany, #72706400) using an analytical balance. Each tube was weighed with and without the biopsy. For MSC-isolation, biopsies were transferred into 50ml tubes (Greiner Bio-One, Frickenhausen, Germany, #227261) and incubated for 90min at 37°C in 5ml of preheated digestion medium. Digestion medium consisted of 5ml DMEM + 4.5g glucose + GlutaMaxI (Gibco, #31966-021), 10mg (0.2% wt/vol) collagenase I (Wako, #037-17601) and 250µg (0.005% wt/vol) DNase I (Sigma-Aldrich Chemie, #DN25-100MG). During the incubation period the sample was gently swiveled every 30min. After 90min, 20ml PBS plus 5% FCS was added to stop the enzymatic reaction. The sample was mixed gently in order to release the cells. The single cell suspension was transferred to a separate tube. After centrifugation (300g, 5min at room temperature) the supernatant was discarded and the pellet resuspended in PBS plus 5% FCS.

**Functional detection of MSC**

Functional detection of MSC was carried out by performing CFU-F assays using MSC Expansion Media (Miltenyi Biotec, Bergisch Gladbach, Germany, #130-091-680). TNC from trephine biopsies were plated in a density of 4 x 10^4^ per flask (Greiner Bio-One, Frickenhausen, Germany, #690160). Colony numbers are reported in relation to TNC or trephine’s weight.

**Ex vivo expansion and growth kinetics of MSC**

MSC were cultured in DMEM (Gibco, #21885-025) + 10% FCS in cell culture flasks (Greiner Bio-One). Medium exchanges were carried out every 5 days. In passage 0 MSC were cultured until confluence. For passaging, MSC were detached by trypsinization.

To determine growth kinetics 2 x 10^4^ MSC were seeded in 12-well plates and cultured for 7 days in DMEM (Gibco, #21885-025) + 10% FCS. Cells were counted using a hemocytometer (Neubauer Laboroptik). Growth kinetics are given as number of cell doublings within these 7 days.

**Osteogenic and adipogenic differentiation**

In brief, 5 x 10^4^ MSC in passage 2 were incubated with the supplements for osteogenic (0.1µM dexamethasone, 0.2mM ascorbate-2-phosphate and 10mM beta-glycerol phosphate) or adipogenic (0.5mM 1-methyl-3-butylisoxanthine, 1µM dexamethasone, 100µM indomethacin, 10µM insulin) differentiation. All reagents for differentiation induction were purchased from Sigma-Aldrich.

Osteogenic differentiation was assessed by determination of activity of tissue nonspecific alkaline phosphatase (TNAP) given as mU/mg protein in differentiated wells related to not differentiated wells.

Adipogenic differentiation was assessed by Oil Red O staining and quantified with the help of AIDeveloper an open source software tool (<https://github.com/maikherbig/AIDeveloper>), which was engaged to train a convolutional neural net (CNN) to detect differentiated adipocytes. The extent of adipogenic differentiation is given in percentage of tiles containing differentiation sites. Further details are provided elsewhere (1,2).

**Immunosuppressive potential of MSC**

To determine the immunosuppressive capacity of MSC from patients after allogeneic HCT, irradiated cultured MSC were cocultured with peripheral blood mononucleated cells (PBMCs) from healthy donors in a lymphocyte mitogen stimulation assay. Blood samples were obtained from healthy donors with informed consent. PBMCs were prepared by Ficoll‐Hypaque (Biochrom, #L6145) density centrifugation. In detail, MSC were first irradiated with a dose of 30 Gray in suspension in tubes by using a Caesium^137^ source (Gammacell 3000 Elan device, Best Theratronics, Ottawa, Canada) and then seeded in round-bottomed 96-wells with a density of 5 × 10^3^ cells per well. After 24h PBMCs were added at a PBMC:MSC ratio 20:1 (1 × 10^5^ cells per well) and then stimulated with anti-CD3/CD28 Dynabeads (Gibco, USA) for 6 days at 37°C/5%CO2. Culture medium consisted of RPMI 1640 supplemented with 2mM L‐glutamine, 10mM sodium pyruvate, 1% nonessential amino acids, 100μg per ml penicillin, 100μg per ml streptomycin, and 10% FCS (all from Biochrom). 3H‐thymidine (1 μCi, Hartmann Analytic, Braunschweig, Germany) was added to each well for the last 18 hours of culture. Cells were harvested and proliferation was assessed as a measurement of 3H-thymidine incorporation into DNA as determined with the MicroBeta 2 (PerkinElmer, Rodgau, Germany).

**Long-term culture – initiating cell (LTC-IC) and colony-forming cell (CFC) assay**

The ability to support hematopoiesis was tested by LTC-IC und CFC assay. For LTC-IC layers of passage 2 MSC were generated by seeding 1.5 x 10^4^ passage 1 MSC in DMEM + 10% FCS in 24 well plates (three wells/patient). After 2 days medium was changed to long-term initiating-cell medium (Stem Cell Technologies, #05150) containing 10^-6^ M hydrocortisone and 1 x 10^3^ HSC of a healthy donor were added to each well. For all experiments HSC of the same donor were used. After one, two and three weeks total cell layers were photographed using a Celigo S (Nexcelom Bioscience, Lawrence, Massachusetts, USA) and scored for cobblestone area-forming cells (CAFC) defined as phase-dark cells underneath the feeder layer. Medium exchange was done every week before counting of CAFC. Besides counting total area of CAFCs were analyzed using ImageJ digital analysis.

For CFC assay one coculture of the LTC-IC was detached by trypsinization after two weeks. 1.5 x 10^4^ of these harvested cells (mixture of MSC and CAFC) were immediately distributed on three wells of a square bioassay dish (Greiner bio-one, #688102) and cultured in Stem MACS™ HSC-CFU complete with erythropoietin (Miltenyi Biotec, #130-091-280) for two weeks. Analysis of CFU (CFU-GEMM, CFU-GM and BFU-E) was done with a STEMvisionTM (STEMCELL TechnologiesTM).

Detached cocultures were also stained for CD45 (anti-CD45 PE from eBiosciences, #12-0459-42) and CD34 (anti-CD34 AF647 from BioLegend, #343508) for multicolor flow cytometry analysis using a MACS Quant (Miltenyi Biotec) in order to determine the absolute numbers of CD45^+^CD34^+^ and CD45^+^CD34^-^ cells.

**Multicolor flow cytometry analysis of freshly isolated, uncultured MSC**

Freshly isolated, uncultured MSC were detected and characterized by staining TNC from BM aspirates and trephine biopsy specimens with the following human monoclonal antibodies: anti-CD235a FITC (eBioscience, #11-9886-42), anti-CD45 V500 (BD Biosciences, #560777), anti-CD90 APC (eBiosciene, #7-0909-41), anti-CD106 PE-Cy7 (eBioscience, #25-1069-41), anti-CD146 BV421 (BioLegend, #361003) and anti-CD271 PE (eBioscience, #12-9400-41). Finally, cells were resuspended in propidium iodide (PI) solution for discrimination of dead cells.

MSC were defined as PI-CD235a-CD45-CD271+. For an example of the gating strategy see Fig. S1. Quantity of phenotypically detected uncultured MSC is given as a MSC/leucocyte ratio (PI-CD235a-CD45+). The staining was performed at 4°C for 30min. Analysis was performed on an ARIA II (BD Biosciences, San Jose, California, USA). Data were analyzed using FlowJo software (Tree Star, Ashland, Oregon).

**Histology and immunochemistry**

Tissue sections were deparaffinized in xylene (3 x 10min, VWR International) and hydrated by graded washes of ethanol (Berkel AHK) to water (B. Braun). Sections were boiled in citrate buffer (Zytomed Systems) at pH 9 for antigen retrieval. Thereafter, tissues were incubated with monoclonal mouse aCD271 antibody (abcam, #ab3125) overnight at 4°C. CD271^+^ cells were visualized by the EnVision G|2 Doublestain System (alkaline phosphatase^+^ [AP], DAKO/Agilent, #K5361) according to the manufacturer’s instructions. All tissue sections were counterstained with Mayers hematoxylin (Merck). Measurement and analysis of tissue slides was performed with Vectra 3.0 automated Quantitative Pathology Imaging System and inForm-Software from PerkinElmer. Based on hematoxylin staining of nuclei, user-trained machine learning algorithms of inForm-Software defined cellularized and non-cellularized areas of up to 100 regions of interest (ROI) for each section. Based on these data, the whole area of cellularized BM tissue was calculated in mm^2^. Due to strong branching of CD271^+^ cells, it was not possible to calculate absolute MSC numbers. Instead the AP^+^ area was calculated in relation to the total cellularized area with the help of the software tools ImageJ and R.

**Detection of BM infiltrating immune cells**

Immune Cells were analyzed in TNC from BM aspirates after staining with the following human monoclonal antibodies:

Dendritic cells (DC): anti-CD45 V500 (BD Biosciences, #560777), anti-CD3 BV421 (BD Biosciences, #562426), anti-CD19 BV421 (BD Biosciences, #562426), anti-CD11c BV605 (BD Biosciences, #563929), anti-HLA-DR APC-H7 (BD Biosciences, #641411), anti-CD1c AlexaF700 (BioLegend, #331530), anti-CD141 PE (Miltenyi Biotec, Bergisch Gladbach, Germany, #130-091-680), anti-Slan FITC (Miltenyi Biotec, Bergisch Gladbach, Germany, #130-093-027), anti-CD123 PE-Cy7 (BioLegend, #306010) and anti-CD303 APC (Miltenyi Biotec, Bergisch Gladbach, Germany, #130-090-905).

B cells and T cell memory subsets: anti-CD45 V500 (BD Biosciences, #560777), anti-CD19 FITC (Biozol, #DAK-F0768), anti-CD3 PerCP-Cy5.5 (BD Biosciences, #332771), anti-CD4 eFluor450 (eBioscience, #48-0047-42), anti-CD8 eFluor780 (eBioscience, #9047-0087), anti-CD197 FITC (BD Biosciences, #561271), anti-CD45RA (BD Biosciences, #555489), anti-CD95 (eBioscience, #17-0959-42) and anti-CD28 PE-Cy7 (BD Biosciences, #555489).

Regulatory T cells: anti-CD45 V500 (BD Biosciences, #560777), anti-CD3 APC-H7 (BD Biosciences, #641415), anti-CD4/CD25 FITC/APC (eBioscience, #22-0425-73), anti-CD127 PerCP-Cy5.5 (BD Biosciences, #560551), anti-CD45RO PE (BD Biosciences, #555493), anti-CD45RA eFluor450 (eBioscience, #48-0458-42) and anti-FOXP3 PE-Cy7 (eBioscience, #25-4776-42).

Staining was performed at 4°C for 30min. Analysis was performed on an LSR II (BD Biosciences) Gating strategies are depicted within the according figures in the results section. Data were analyzed using FlowJo software (Tree Star, Ashland, Oregon).

**Statistical analysis**

Statistical analysis was performed using GraphPad Prism software version 9.0 (GraphPad Software, La Jolla, CA, USA). Data are presented as median with ranges. Mann-Whitney test was used for statistical analysis of continuous variables (without Gaussian distribution). A p-value of less than 0.05 was regarded as statistically significant. For multivariable analysis of MSC numbers a linear regression model was fitted. MSC numbers were log-transformed to achieve compatibility with the normality assumption. Estimates were then transformed back to the original scale.

# Supplementary Figures and Tables

## Supplementary Figures


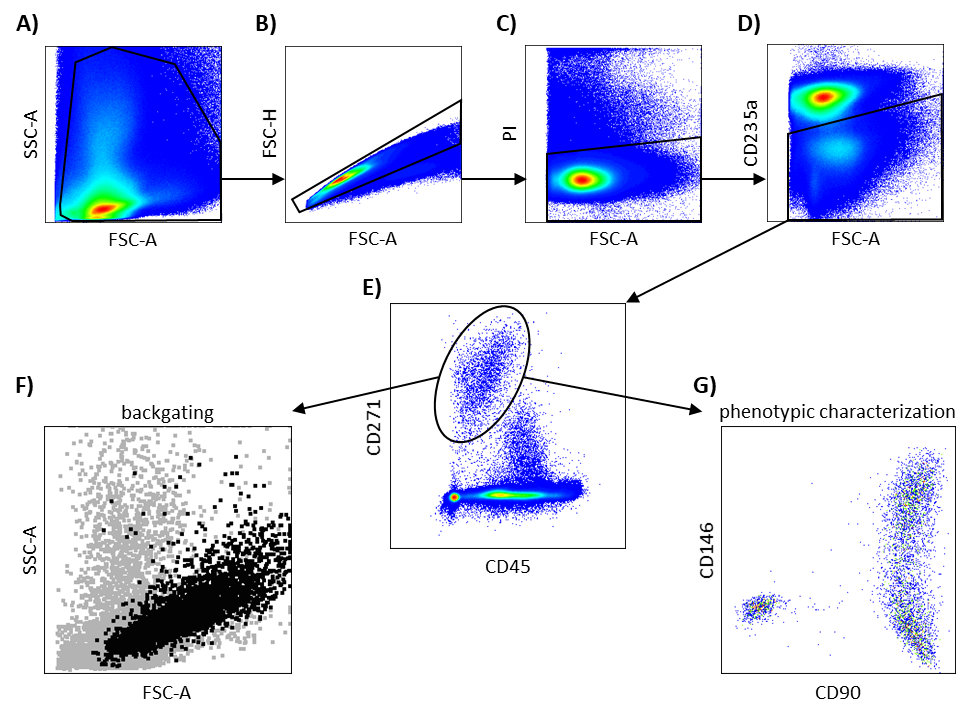


**Figure S 1 Flow cytometric detection of uncultured MSC.**

A) Forward versus side scatter (FSC vs. SSC) is used to identify cells of interest according to size and granularity/complexity and to exclude debris. B) Forward scatter area (FSC-A) versus forward scatter height (FSC-H) is used to exclude doublets. C) Labeling with propidium iodid (PI) is used to exclude dead cells. D) Since CD235a is restricted to the erythroid lineage, it is used to exclude erythrocytes and main erythroid precursors. E) Within CD235a^-^ living singlets uncultured MSC were defined as being CD45^-^CD271^+^. E) Backgating of detected uncultured MSC according to size (FSC-A) and granularity/complexity (SSC-A) to confirm the gating strategy. F) Phenotypic characterization of uncultured MSC, in this example displayed according to their surface expression of CD90 and CD146


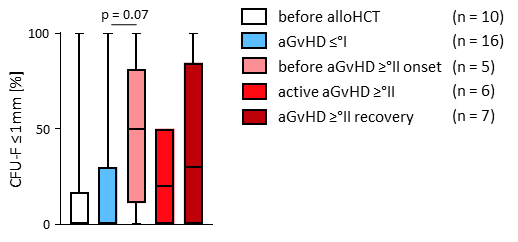
**Figure S2 Proportion of small colonies (≤ 1mm) in CFU-F assays produced by BM aspirate MSC P0.** The diagrams show boxplots with whiskers from minimum to maximum. Mann-Whitney test was used for statistical analysis. n, number

**References**

1. Krüger T, Middeke JM, Stölzel F, Mütherig A, List C, Brandt K, et al. Reliable isolation of human mesenchymal stromal cells from bone marrow biopsy specimens in patients after allogeneic hematopoietic cell transplantation. Cytotherapy. 2020;22(1):21–6.

2. Kräter M, Abuhattum S, Soteriou D, Jacobi A, Krüger T, Guck J, et al. AIDeveloper: Deep Learning Image Classification in Life Science and Beyond. Adv Sci. 2021;8(11):1–12.

**
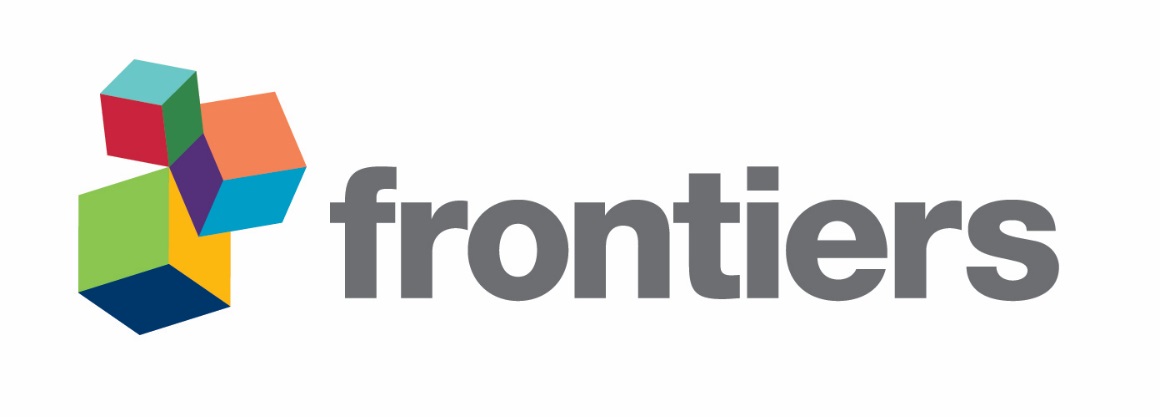
**
