# Supplementary figures and images for: Perturbations of mesenchymal stromal cells after allogeneic hematopoietic cell transplantation predispose for bone marrow graft-versus-host-disease
Source: Front Immunol. 2022 Oct 12;13:1005554. doi: 10.3389/fimmu.2022.1005554 (PMC9599394; doi:10.3389/fimmu.2022.1005554)

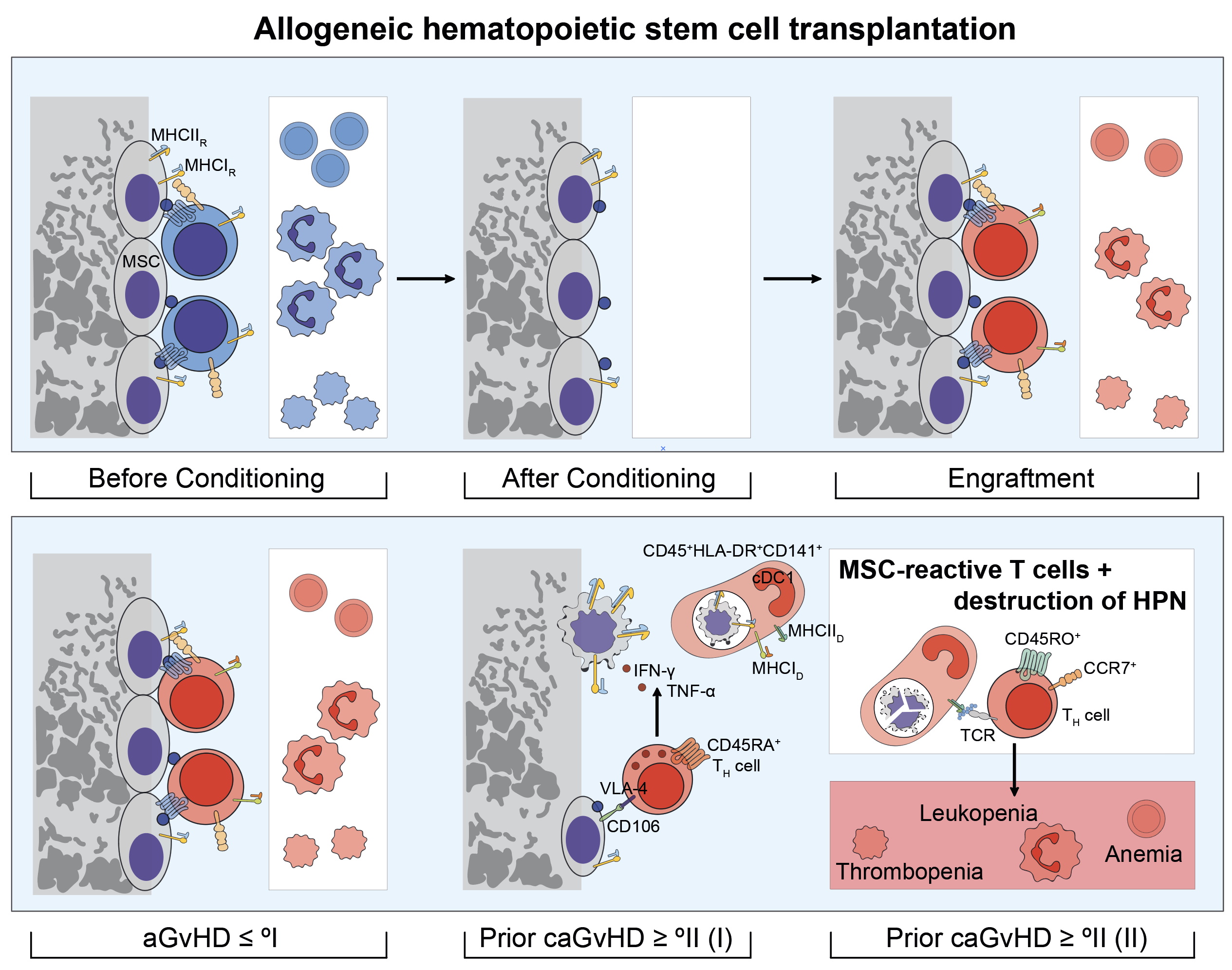

Supplement: Supplementary file 2 [file Image_1.jpeg]
